# Bed bug control with various dusts: Efficacy comparison between silicon dioxide, diatomaceous earth, and Sommières earth[Fn FN1]

**DOI:** 10.1051/parasite/2024040

**Published:** 2024-07-24

**Authors:** Halilou Almou Oumarou, Harouna Tahirou Hima, Jean Michel Berenger, Grégory Michel, Olivier Grauby, Philippe Parola, Christelle Pomares, Pascal Delaunay

**Affiliations:** 1 Service de Parasitologie-Mycologie, Hôpital de l’Archet, Centre Hospitalier Universitaire de Nice 151, route de Saint Antoine de Ginestière, CS 23079 06202 Nice cedex 3 France; 2 Université d’Aix Marseille, IRD, SSA, AP-HM, VITROME 19-21 Boulevard Jean Moulin 13385 Marseille Cedex 05 France; 3 Inserm U1065, Mediterranean Center for Molecular Medicine (C3M), Université de la Côte d’Azur 151 route Saint Antoine de Ginestière BP 2 3194 06204 Nice France; 4 Université d’Aix Marseille et Centre Interdisciplinaire de Nanoscience de Marseille-CNRS Campus de Luminy Case 913 13288 Marseille Cedex 9 France; 5 Institut Hospitalo-Universitaire Méditerranée Infection 19-21 Boulevard Jean Moulin 13385 Marseille Cedex 05 France

**Keywords:** *Cimex lectularius*, Bed bugs, Diatomaceous earth, Sommières earth, Silicon dioxide, Dust

## Abstract

Bed bugs are considered a major public health problem in industrialized countries. Usually, bed bug infestations are managed using a combination of physical and chemical methods. In recent years, new strategies for bed bug control have emerged, particularly the use of dusts like diatomaceous earth and silicon dioxide. However, in Europe, the use of silicon dioxide is restricted to professional, while diatomaceous earth can be harmful to the lungs. This study aimed to assess bed bug mortality rates associated with Sommières earth, green clay, talc, and sodium bicarbonate compared to silicon dioxide and diatomaceous earth from a pest management company, diatomaceous earth for litter conditioner, and diatomaceous earth from a supermarket. We tested permanent exposure, short exposure, horizontal transfer and repellent effect on two bed bug colonies. Sommières earth demonstrated efficacy ranging from 75% to 100% in permanent and short exposures, similar to the efficacy of diatomaceous earth from the pest management company. On the contrary, diatomaceous earth for litter conditioner and diatomaceous earth from a supermarket, green clay, talc, and sodium bicarbonate were found to be ineffective. This study demonstrates, for the first time, the efficacy of Sommières earth against bed bugs, but also highlights the variability in efficacy of diatomaceous earths on bed bugs depending on their quality.

## Abstract (fr)

Les punaises de lit sont considérées comme un problème de santé publique majeur dans les pays industrialisés. Habituellement, les infestations de punaises de lit sont gérées en utilisant une combinaison de méthodes physiques et chimiques. Ces dernières années, de nouvelles stratégies de lutte contre les punaises de lit ont vu le jour, notamment l’utilisation de poudres comme la terre de diatomées et le dioxyde de silicium. Cependant, en Europe, l’usage du dioxyde de silicium est réservé aux professionnels tandis que la terre de diatomées peut être nocive pour les poumons. Cette étude visait à évaluer les taux de mortalité des punaises de lit associés à la terre de Sommières, à l’argile verte, au talc et au bicarbonate de sodium par rapport au dioxyde de silicium, à la terre de diatomées d’une entreprise de lutte antiparasitaire, à la terre de diatomées pour conditionneur de litière et à la terre de diatomées d’un supermarché. Nous avons testé l’exposition permanente, l’exposition courte, le transfert horizontal et l’effet répulsif sur deux colonies de punaises de lit. La terre de Sommières a démontré une efficacité allant de 75% à 100% en exposition permanente et courte, similaire à l’efficacité de la terre de diatomées d’une entreprise de lutte antiparasitaire. Au contraire, la terre de diatomées pour conditionneur de litière et la terre de diatomées d’un supermarché, l’argile verte, le talc et le bicarbonate de sodium se sont révélés inefficaces. Cette étude démontre, pour la première fois, l’efficacité de la terre de Sommières contre les punaises de lit mais met également en évidence la variabilité de l’efficacité des terres de diatomées sur les punaises de lit en fonction de leur qualité.

## Introduction

Bed bugs are insects classified within the family Cimicidae and the genus *Cimex* [2, 13, 28]. In the context of human nuisances, the term “bed bug” is associated with two species: *Cimex lectularius* Linnaeus, 1758 and *Cimex hemipterus* Fabricius, 1803, found in temperate and tropical/equatorial regions, respectively [2]. These insects exclusively feed on blood and undergo incomplete metamorphosis, progressing through five stages before the adult stage. Their size can range from 1 to 7 mm [9]. Bed bugs represent a challenge in household pest control due to their preference for dark, narrow spaces that are often difficult to access. The annual resurgence rate of the global bed bugs population is estimated to range from 100 to 500% [20]. Eradicating infestations within a dwelling or on a larger scale within a building requires several actions, employing mechanical (cleaning, laundry, and vacuum), physical (heat), and chemical methods. Mechanical and physical methods have the advantages of being non-toxic and can be repeated without the concern of resistance. Additionally, the risk of widespread dispersion remains low, except in cases of extensive thermal control targeting entire residences or buildings [23]. However, the drawback of mechanical methods lies in their limited persistence. A single fertilized female or a few overlooked eggs can lead to persistence of the infestation [29]. Although not obligatory, the use of insecticides is common, and the presence of these chemical agents can contribute to effective control. Unfortunately, toxicity and resistance have been documented across all chemical classes of insecticides [12]. The development of resistance stands as one of the factors contributing to the resurgence of bed bug infestations observed worldwide [15]. In addition to documented resistance to the chemical approach, the repellent properties of these compounds can inadvertently foster bed bug dispersion [4, 8].

In recent years, a bed bug control strategy employing so-called “natural” dusts has gained popularity. These dusts display potent absorption properties, as observed in silicon dioxide (SD), or a combination of absorption and abrasion actions found in diatomaceous earth (DE) [3, 18, 19, 34, 35]. In detail, diatomaceous earth adheres to the bed bug’s body and damages the protective waxy layer of the bed bug cuticle through absorption and abrasion. These particles enter the body of bed bugs and get stuck between the joints of their exoskeletons. When the bed bug moves, these sharp particles physically cut into the bed bugs organs. Therefore, this leads to the loss of water from the bed bug body and ultimately death [5]. When silicon dioxide is in contact with the insect, it is absorbed by cuticular lipids, resulting in considerable injury and insect death by desiccation [6]. Silicon dioxide [21] and diatomaceous earth are the reference standards, having demonstrated their effectiveness against bed bugs through empirical testing [3, 33]. However, in Europe, the use of silicon dioxide is restricted to professionals, while diatomaceous earth can be harmful to the lungs [1]. The latter has the ability to act through horizontal transfer, allowing the dust to be delivered and disseminated thanks to the interactions between conspecifics [3]. Besides the efficacy of silicon dioxide and diatomaceous earth, we assessed “consumer-grade” dusts such as Sommières earth (SE), green clay (GC), sodium bicarbonate (SB), and talc dusts (Ta) known for their significant adsorption and absorption properties, but without abrasion properties. These dusts are commonly used to absorb liquids from various surfaces such as fabric, wood, and tiles, for stain removal due to their absorption capacity. We aimed to study green clay, easily available in supermarkets, known for its cost-effectiveness, non-toxic nature, and absorption capacities [4]. We included talc dust in the experiments, recognized for its absorbent properties and sometimes suggested in bed bug traps [16]. Lastly, sodium bicarbonate dust, occasionally recommended as a control measure, is found in some website resources associated with the terms “sodium bicarbonate and bed bugs” [24].

Thus, our study assessed the mortality rates induced by four dust, i.e., Sommières earth, green clay, talc dust, and sodium bicarbonate in comparison with two reference dusts widely recognized for bed bug control: silicon dioxide and diatomaceous earth.

## Materials and methods

### Bed bugs

#### Origins

Two strains of *Cimex lectularius* bed bugs were used: one strain, labeled as “Colony 1”, collected from a dwelling in Nice, France (22/06/2022) by the Laboratory of Parasitology and Mycology, Nice teaching hospital; a second strain, named “Colony 2”, obtained from a dwelling in Marseille, France (07/07/2020) and collected by the Laboratory of Medical Entomology, Marseille “Institut Hospitalier Universitaire”. For the experimental conditions described below, 1200 bed bugs were used.

#### Breeding conditions

**Figure 1:**
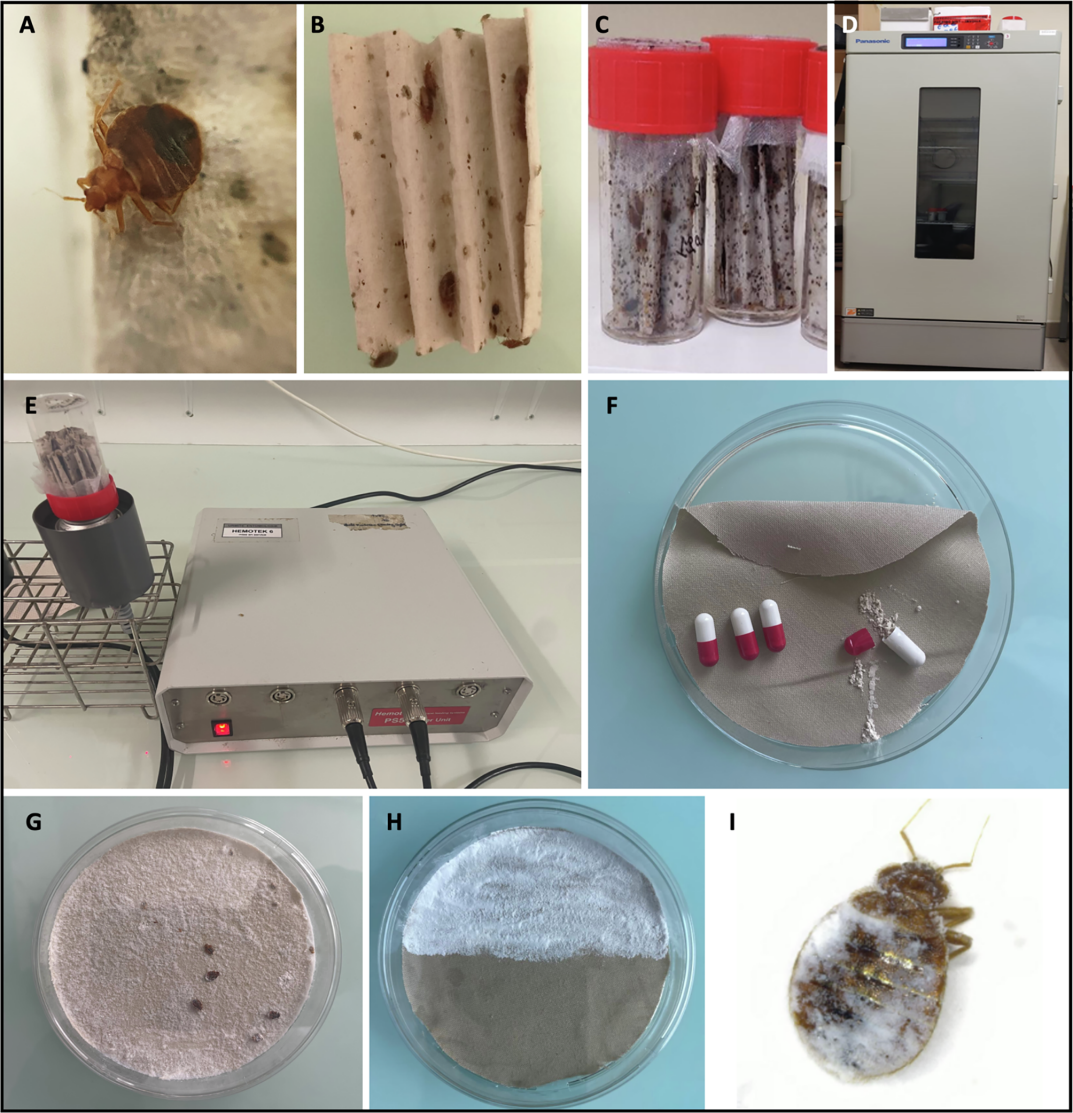
Material and methods. (A) Bed bug (*Cimex lectularius*). (B) Bed bug on folded paper support. (C) Bed bugs and paper supports in plastic containers. (D) Incubator at 24 ± 1 °C, with 56% relative humidity photoperiod 12–12 h. (E) Blood feedings twice a week on “Hemotek” feeding system. (F) Dust testing equipment: Petri dish, cotton fabric at the bottom, capsule filled in with dust with standardized 0.21 mL volume. (G) Experimental conditions: the dust is distributed evenly, and bed bugs are placed on the dusted support. (H) Repellent experiment: bed bugs were placed on the central axis between the dusted and non-dusted areas. (I) A dead bed bug after exposure to SD.

Both strains were maintained at the Laboratory of Parasitology Mycology of the teaching hospital of Nice. Bed bugs were housed in 50 mL plastic containers sealed with screw lids. Folded papers placed inside the containers served as shelter and oviposition sites (Fig. 1A–C). The colonies were maintained at a temperature of 24 ± 1 °C in an incubator, with relative humidity of 56% and a 12-h light-dark photoperiod (Fig. 1D). Blood feedings were performed twice a week using the “Hemotek” feeding system (Hemotek Ltd, Great Harwood, United Kingdom) (Fig. 1E), with packed red blood cells supplied by the “Établissement Français du Sang” [32].

### Dusts

A total of eight dusts were tested on bed bugs: silicon dioxide (SD) CimeXa (Rockwell Labs Ltd, Kansas City, MO, USA), diatomaceous earth from a pest management company (DE-pro) (FOR BUG^®^ “P insectosec” by EDIALUX^®^, Mâcon, France), diatomaceous earth commercialized for litter conditioner (DE-pet) (Protecta^®^, Huddersfield, UK), diatomaceous earth from the supermarket for insect control (DE-hom) (Starwax^®^, Wasquehal France), Sommières earth (SE) (HugeDomais^®^) green clay (GC) (Naturado^®^), talc (Ta) (supermarket), and sodium bicarbonate (SB) (supermarket). All of these dusts were purchased to be tested under our experimental conditions.

### Standardization of dust deposition

To account for variations in dust density, we standardized the delivered volume of each dust by employing pharmaceutical capsules of 0.21 mL capacity filled by the Pharmacy of the Nice teaching hospital. Between 1 and 8 capsules of each type of dust were spread onto Petri dishes (Fig. 1F).

### Experimental conditions

The experiments were conducted in plastic Petri dishes (height: 1.5 cm, diameter: 8.5 cm) with a surface area of 56.7 cm². The bottom of the Petri dishes was coated with beige 100% cotton fabric, depending on the experimental conditions, covered or not by dusts (Fig. 1F, G). Bed bugs were introduced into these dishes. Two replicates were performed for each experimental condition. The bed bugs were starved for a week before each experiment. For each experimental condition, non-exposed bed bugs corresponding to a Petri dish free of dust coated with fabric were used as controls.

#### Mortality assessment: “permanent exposure”

The following volumes of each dust were distributed out at the bottom of Petri Dishes: 0.21 mL (1 capsule); 0.42 mL (2 capsules); 0.84 mL (4 capsules), and 1.68 mL (8 capsules). Ten bed bugs (2 females, 2 males, and 6 nymphs) were placed in the Petri dishes previously prepared with the dusts and continuously exposed for 10 days (Fig. 1G). Mortality was assessed daily. The eight dusts tested were: SD, DE-pro, DE-pet, DE-hom, SE, GC, Ta, and SB.

#### Mortality assessment: “short exposure”

Ten bed bugs (2 females, 2 males, and 6 nymphs) were placed in Petri dishes covered with dust. After a 10-min exposure, the bed bugs were transferred to dust-free Petri dishes coated with fabric. Mortality was monitored daily for 10 days (Fig. 1G). For this experiment, the volume of dusts resulting in 100% mortality on day 10 during permanent exposure was used plus DE-hom dust.

#### Mortality assessment: “horizontal transfer”

Four dusts: SD, DE-pro and SE due to their proven effectiveness from both long and short exposures, and DE-hom because of its presumed widespread use, were used for this experiment. For each dust, a volume of 1.68 mL was spread in a Petri dish, and four adult bed bugs (2 females and 2 males) were exposed for 10 min. Following the exposure period, the adult bed bugs were transferred to dust-free Petri dishes, each containing six bed bug nymphs of stages 3, 4, and 5. Consequently, each Petri dish comprised 10 bed bugs (including 2 females and 2 males exposed to dust and six nymphs not exposed). A negative control was employed consisting of a Petri dish containing 10 non-exposed bed bugs (2 females, 2 males and 6 nymphs). Bed bug mortality was recorded daily for each dust over a period of 10 days.

#### Repellent effect evaluation

All dusts were assessed for their repellent action. The surface of the Petri dishes was evenly divided into two sections: one half with dust (half of the volume used in the previous experiments) and the other without dust (Fig. 1H). Ten bed bugs (2 females, 2 males, and 6 nymphs) were placed on the central axis between the two areas. Bed bug behavior was observed every 10 min for 1 h. Percentage repellency (PR) was calculated using the formula by Kakati et al*.* [25]. PR = [(Nc−Nd)/(Nt)] × 100; Nc = total number of bed bugs in a dust-free area; Nd = total number of bed bugs on a dusted area, Nt = total number of bed bugs. For PR, the lower the number, the lower repellent action. Repellent effect was tested in addition to the mortality assessment in order to ensure that there will be no behavioral resistance to dusts [22].

### Structural study of dusts

Structural analysis of the dusts was conducted by the “Centre Interdisciplinaire de Nanoscience de Marseille” Laboratory, Aix-Marseille University. The equipment used was a Model JEOL JSM-7900F scanning electron microscope, with magnifications ranging from 700 to 50,000, at accelerating voltages of 5 and 15 kV.

### Statistical analysis

A log-rank (Mantel-Cox) test was performed using GraphPad Prism version 9.0.0 for Windows (GraphPad Software, San Diego, CA, USA, www.graphpad.com). A difference was considered statistically significant when the *p* value was <0.05.

## Results

The experiments using 0.21 mL (1 capsule), 0.42 mL (2 capsules), and 0.84 mL (4 capsules) of each dust did not yield conclusive results, showing either no or very limited mortality. Therefore, these volumes were not used. The application of 1.68 mL of dust (8 capsules) yielded usable mortality results and was selected for the permanent exposure, short exposure, and horizontal transfer experiments.

### Permanent exposure

**Figure 2:**
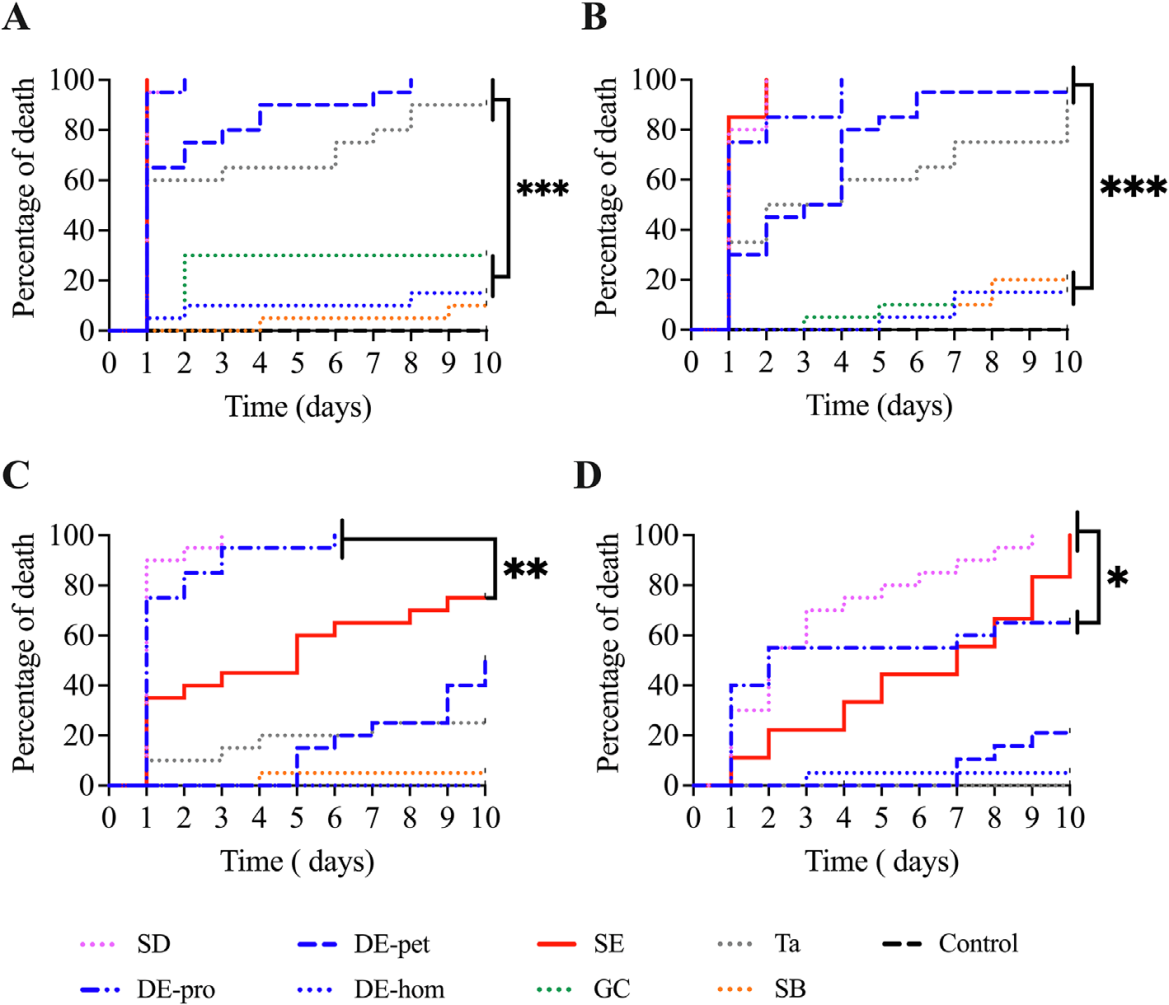
Permanent and short exposure studied over 10 days. (A, B) Permanent exposure tested on Colony 1 and 2 exposed to SD, DE-pro, DE-pet, DE-hom, SE, GC, Ta, SB, and control condition. (C, D) Short exposure tested on Colonies 1 and 2 exposed to SD, DE-pro, DE-pet, DE-hom, SE, Ta, and control condition.

Concerning the eight tested dusts, 100% mortality was observed with SD, DE-pro, DE-pet, SE, and Ta on day 10 for both colonies 1 and 2 (Table 1) (Fig. 2A and B). SD and SE achieved 100% mortality on the first and second days for colony 1 (Fig. 2A) and colony 2 (Fig. 2B), respectively. With DE-pro, colony 1 exhibited 100% mortality on the second day, whereas colony 2 achieved this on the fourth day. For DE-pet and Ta, 100% deaths occurred between the eighth and tenth days for both bed bug colonies. The fastest-acting dusts, resulting in over 90% bed bug mortality by day 3, were SD, DE-pro, and SE. A significant difference (*p* = 0.0001) in bed bug mortality was observed over a 10-day period between the dusts SD, DE-pro, DE-pet, SE, Ta and DE-hom, CG, SB, across both bed bug colonies. DE-hom, GC, and SB displayed less than 30% mortality for colony 1 (Fig. 2A) and less than 20% for colony 2 on days 10 (Fig. 2B). No mortality was observed in the experimental control conditions (Table 1).

**Table 1:** Results of permanent exposure, short exposure, and horizontal transfer on the two bed bug colonies.

		Mortality on colony 1 (%)				Mortality on colony 2 (%)			
		Day 1	Day 2	Day 6	Day 10	Day 1	Day 2	Day 6	Day 10
Permanent exposure	Silicon dioxide	100	100	100	100	80	100	100	100
	Diatomaceous earth pest management company (DE-pro)	95	100	100	100	75	85	100	100
	Diatomaceous earth litter conditioner (DE-pet)	65	75	90	100	30	45	85	100
	Diatomaceous earth supermarket insect control (DE-hom)	5	10	10	15	0	0	5	15
	Sommières earth	100	100	100	100	85	100	100	100
	Green clay	5	30	30	30	0	5	10	15
	Talc	60	60	65	100	35	50	60	100
	Sodium bicarbonate	0	0	5	10	0	0	10	15
	Control	0	0	0	0	0	0	0	0
Short exposure	Silicon dioxide	90	95	100	100	30	55	80	100
	Diatomaceous earth pest management company (DE-pro)	75	85	100	100	40	55	55	65
	Diatomaceous earth litter conditioner (DE-pet)	0	0	20	50	0	0	0	20
	Diatomaceous earth supermarket insect control (DE-hom)	0	0	0	0	0	5	5	5
	Sommières earth	35	40	60	75	10	20	45	95
	Talc	10	15	20	25	0	0	0	0
	Control	0	0	0	0	0	0	0	0
Horizontal transfer	Silicon dioxide	35	45	70	95	55	75	85	100
	Diatomaceous earth pest management company (DE-pro)	45	55	75	85	45	55	75	95
	Diatomaceous earth supermarket insect control (DE-hom)	0	20	25	30	5	10	10	10
	Sommières earth	0	0	20	55	0	10	10	70
	Control	0	0	0	10	0	0	0	0

### Short exposure

Among the six tested dusts, SD exhibited 100% mortality by day 10 for both colonies 1 and 2 (Table 1) (Fig. 2C and D). By day 10, DE-pro demonstrated 100% mortality for colony 1 and 65% mortality for colony 2. SE displayed over 70% mortality for colonies 1 (75%) and 2 (95%) (Fig. 2C and D). DE-pet, DE-hom, and Ta showed limited mortality (≤50%) for colonies 1 and 2 by day 10. Unlike to permanent exposure, only SD resulted in over 90% mortality, but solely for colony 1 by day 3. Mortality rates varied for DE-pro and SE between colonies, and it took longer for these dusts to achieve at least 60% mortality compared to permanent exposure. Significant differences in mortality rates were observed between SD, DE-pro, and SE for colony 1 (*p* = 0.0003), and between SD, SE, and DE-pro for colony 2 (*p* = 0.0025) over the 10-day period (Fig. 2C and D). No mortality was observed on day 10 in the experimental control conditions (Table 1).

### Horizontal transfer

**Figure 3:**
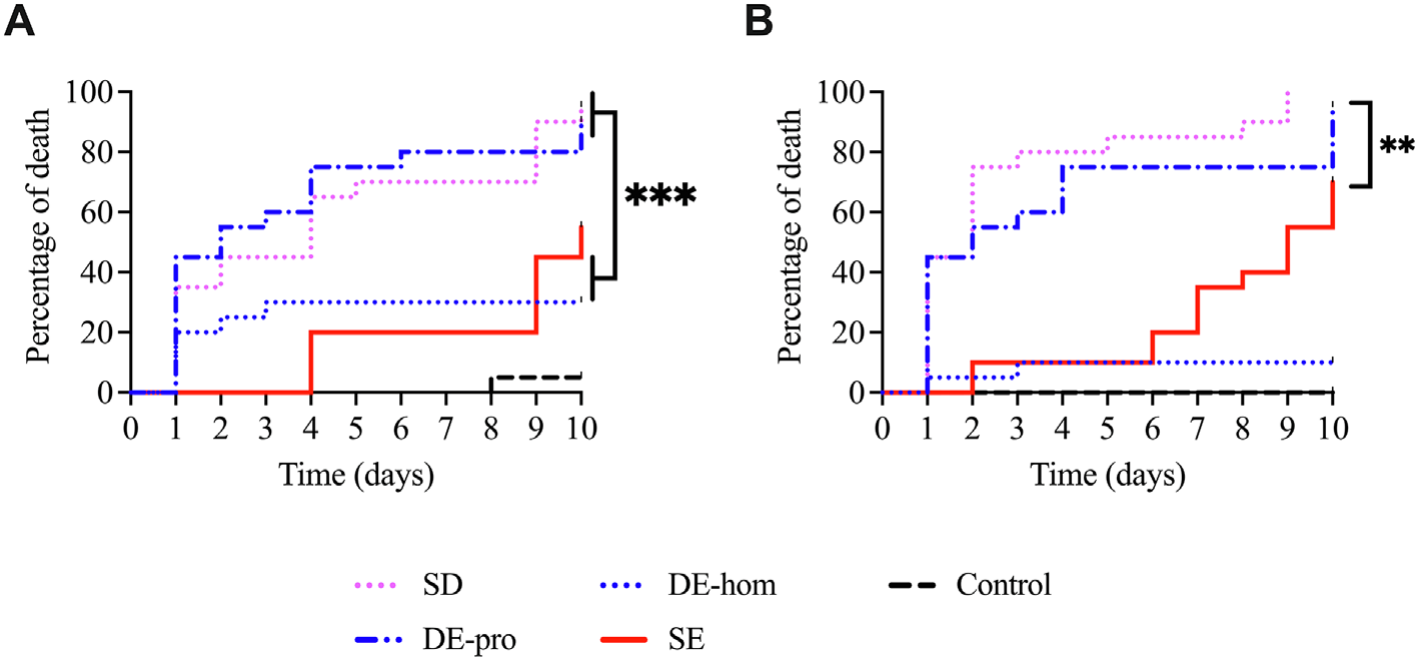
Horizontal transfer studied over 10 days. (A, B) Colonies 1 and 2 exposed to SD; DE-pro, DE-hom, SE, and control condition.

Only SD resulted in 100% mortality by day 9 for colony 2. Mortality rates were over 60% for SD, DE-pro, and SE on both colonies 1 and 2 by day 10, except for SE on colony 1 where mortality was 55% (Table 1) (Fig. 3A and B). By day 10, DE-hom mortality was below 50% at day 10 for both colonies (30% for colony 1 and 10% for colony 2). Mortality exceeding 80% was achieved only by day 10 for SD and DE-pro 10 on both colonies. Significant differences in mortality were observed between SD, DE-pro and SE, DE-hom dusts on colony 1 (*p* = 0.0003) and between SD, DE-pro, SE, and DE-hom dusts for colony 2 (*p* = 0.0037) over a period of 10 days. In the control conditions, a mortality rate of 10% was observed by day 10 for colony 1, while no mortality was observed for colony 2 (Table 1).

### Repellent effect

The percentage of repellent effect (PR) was determined using the formula reported by A. Kakati et al*.* [17]. The PR values of all dusts were below 0. The positive control which is DEET (N,N-diethyl-m-toluamide) at 5% was 100% (Table 2). None of the eight dusts tested showed a repelling effect on bed bugs.

**Table 2:** Repellent effect of dusts on the two bed bug colonies.

	Percentage repellency (%)		Interpretation
	Colony 1 (%)	Colony 2 (%)	
Silicon dioxide	−72%	−45%	Not repellent
Diatomaceous earth pest management company (DE-pro)	−74%	−58%	Not repellent
Diatomaceous earth litter conditioner (DE-pet)	−63%	−33%	Not repellent
Diatomaceous earth supermarket insect control (DE-hom)	−98%	−80%	Not repellent
Sommières earth	−84%	−36%	Not repellent
Green clay	−86%	−73%	Not repellent
Talc	−81%	−56%	Not repellent
Sodium bicarbonate	−94%	−70%	Not repellent
Control (DEET)	100%	100%	Repellent

Percentage repellency was calculated according to the following formula: [(Nc−Nd)/(Nt)] × 100; Nc = total number of bed bugs in a dust-free area; Nd = total number of bed bugs on a dusted area, Nt = total number of bed bugs.

### Morphological and structural study of dusts

**Figure 4:**
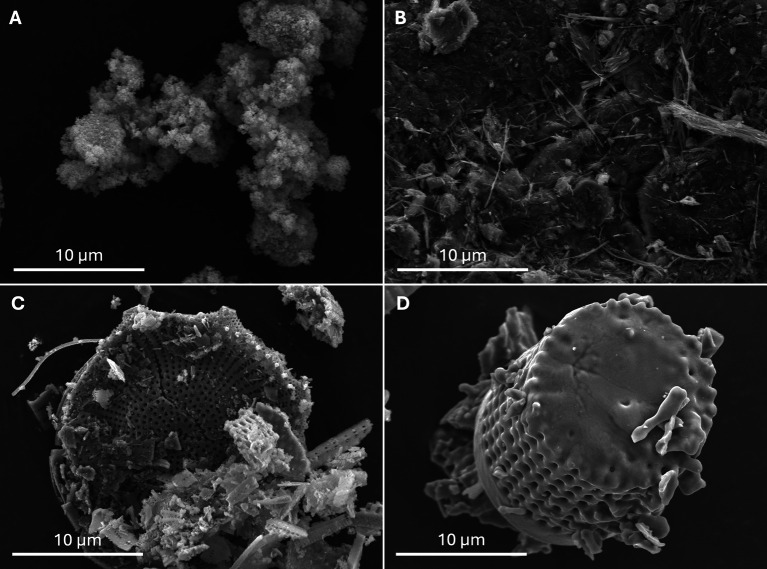
Dusts photographed under a scanning electron microscope. (A) Silicon dioxide dust. Aggregate of nanoparticles (grains smaller than 100 nm) of “amorphous silica”. (B) Sommières earth. Assemblage of fibrous sepiolite crystals. (C) Diatomaceous earth from a pest management company (DE-pro). Frustules (small shells) of “unprocessed” diatoms (unicellular microscopic algae) made of “amorphous silica”. (D) Diatomaceous earth from the supermarket (DE-hom): diatom frustules probably transformed by intense heating, transforming the “initially amorphous silica” into “a cristobalite-type crystalline structure”, suitable for use as a swimming-pool water filter.

The scanning electron microscope images provided detailed views of the morphological and composition (Table 3) characteristics of the examined dust samples. Among the 5 dusts analyzed, SD (Fig. 4A), SE (Fig. 4B), GC, Ta, and SB displayed morphologies consistent with their expected nature. SD appeared as an amorphous compound, while SE exhibited a microfiber structure, contributing to its highly hydrophilic action. The various DE dusts exhibited distinct structures from one another. In their natural state, DE dust corresponds to the accumulation of perfectly preserved skeletons of diatoms, which are hard-shelled aquatic microalgae. These glass micro-skeletons possess abrasive qualities and hydrophilicity through adsorption. DE-pro had the expected morphology, showcasing intact diatoms skeletons (Fig. 4C). Conversely, DE-pet and DE-hom had altered, melted and even fused skeletons (Fig. 4D).

**Table 3:** Composition of the different dusts analyzed.

Dusts	Composition	References
Silicon dioxide	SiO_2_ pure. Amorphous silica, nanometric commonly called colloidal silica	[30]
Diatomaceous earth pest management company (DE-pro)/Diatomaceous earth litter conditioner (DE-pet)/Diatomaceous earth supermarket insect control (DE-hom)	Siliceous skeleton of unicellular algae, diatoms. Diatomaceous earth corresponds to biogenic amorphous silica. The chemical composition is over 80% SiO^2^ plus TiO_2_, Al_2_O_3_, Fe_2_O_3_, MgO, CaO, Na_2_O, K_2_O in various quantity depending on the origin of diatomaceous earth. DE-pet and DE-hom were probably amorphous diatomaceous earth industrially prepared by flux-calcining at high temperature and, consequently, partially crystallized into cristobalite	[14, 26, 27]
Sommières earth	Mainly sepiolite (a mineral from the clay group with a fibrous structure) associated with dolomite, quartz and polygorskite	

## Discussion

The two main methods employed for bed bug control involve chemical methods, through the use of chemical products [37], and physical methods, through the use of devices such as a steam and vacuum cleaners [13]. However, the effectiveness of these methods is limited due to the potential risk of bed bug resistance and dispersal, and the lack of residual protection of physical actions [10]. To date, only a few studies investigating the use of inert dusts against bed bugs have been published [3, 8, 21, 31, 33]. In our study, we compared the efficacy of eight different dusts. The duration required to achieve 100% mortality among bed bugs varied based on the dusts used, the exposure conditions, and the bed bug colony. Our findings demonstrated the effectiveness of three dusts, i.e., SD, DE-pro, and SE in various exposure scenarios: permanent exposure, short exposure, and via horizontal transfer. By day 10, mortality rates ranged from 55% to 100% depending on the colonies and experimental conditions. Conversely, DE-hom, SB, and GC dusts were ineffective, while DE-pet and Ta displayed limited action, predominantly under permanent exposure. The lack of effectiveness of SB on bed bugs contradicts information found on certain websites, that suggest its efficacy in bed bug control [11]. However, our results for SD and DE-pro align with previously published data [3, 8, 33, 36]. These dusts exert their lethal effects on bed bugs through two mechanisms: first, a desiccation process that induces dehydration upon exposure, and second, an abrasive action leading to microcracks on the cuticle, primarily observed with DE (Fig. 1I) [36]. SE also demonstrated a noteworthy lethal effect, likely attributable to desiccation due to the microscopic fiber structure of this dust (Fig. 4).

In our study, the condition referred to as “horizontal transfer” was the experimental setting where 100% mortality was observed in only one dust, SD, and within a single colony. Horizontal transfer is a crucial mechanism in bed bug control, distributing insecticide residues to individuals in difficult-to-reach areas such as cracks, crevices, frames, books, and furniture [3]. Our findings revealed that, under this condition, the three most effective dusts (SD, DE-pro, and SE) exhibited lower efficacy compared to “permanent and short exposure” conditions. Further testing of these dusts in field conditions is warranted to evaluate their effectiveness in this context. The differences observed in the results between the two colonies may be linked to their distinct geographical origins probably related to different genetic backgrounds. In addition, the duration of breeding was different between the two colonies. This time gap might have impacted the microbiota, besides the possibility that bed bugs collected from the field could be more active than those reared in laboratory settings [7]. In our controlled conditions, the oldest laboratory-kept colony, i.e., colony 2 demonstrated a lower mortality rate than colony 1.

We observed significant variation in the efficacy of DE depending on the brand used. DE from a pest management company exhibited high efficacy, whereas diatomaceous earth used as a litter conditioner showed only marginal effectiveness. Additionally, DE sourced from a supermarket was found to be ineffective. This variability in efficacy could potentially be attributed to the structural differences observed among these DE samples. Microscopic analysis revealed that the professional grade (DE-pro) exhibited the typical appearance, whereas the other two samples displayed an atypical shape [14, 26]. Consequently, we hypothesize that the changes from the usual structure of DE may have resulted in the loss of both its desiccating and abrasive properties. This loss, in turn, could reduce its lethal impact on bed bugs, explaining the varying degrees of effectiveness observed among the different DE samples.

These results have inherent limitations. First, they were carried out on a limited number of bed bugs. Second, these findings were not based on “recently” field-collected bedbugs with “natural” digestive and surface microbiota and “natural” behavior. However, conducting such experiments with bed bugs recently collected from the field is difficult to achieve due to the high number of bed bugs required. In addition, bed bugs collected from the field often have a history of insecticide treatments, which could have introduced a bias into the mortality observed during our experiments. However, our laboratory results need to be validated by testing them in the field, and this validation will enable us to decide which dusts to use in current practice. The categorization of degrees of infestation will enable us to make comparisons of dust efficacy in homes with identical degrees of infestation [15]. To date, our results have given an indication of which dusts should be tested under real-life conditions.

Looking ahead, it would be interesting to explore (i) other dusts, (ii) combination of dusts that may exhibit synergistic effects, and (iii) tests for resistance or adaptation to these dusts.

## Conclusion

Among the eight dusts tested, three demonstrated high efficacy against bed bugs: silicon dioxide, diatomaceous earth sourced from a pest management company, and a newly tested dust, Sommières earth. Our study highlights the variability in efficacy observed among different brands of diatomaceous earth in bed bug control, dependent on the quality of the microscopic structure of the dusts, thus rendering their use for this application unreliable.
